# The Split Personality of Beauveria bassiana: Understanding the Molecular Basis of Fungal Parasitism and Mutualism

**DOI:** 10.1128/mSystems.00766-21

**Published:** 2021-08-24

**Authors:** Almudena Ortiz-Urquiza

**Affiliations:** a Department of Biosciences, Faculty of Science and Engineering, Swansea University, Swansea, United Kingdom

**Keywords:** *Beauveria bassiana*, VOCs, cuticular lipids, endophytic growth, fungal toxins, lipid assimilation, lipid hydrolysis, plant-fungus association, saprobe/parasite/mutualist continuum, targeted gene knockout

## Abstract

Fungal pathogenicity toward insects has independently evolved several times, resulting in specialist and generalist pathogens, some of whom have maintained aspects of their previous lifestyles. Being able to grow as an endophyte (engaging in a mutualistic interaction with plants) or saprophyte (recycling nutrients back into the environment), the generalist (broad-host-range) fungus Beauveria bassiana does not need to rely on insect hosts to complete its life cycle. The diverse lifestyles of this fungus, saprophyte, pathogen, and symbiont, provide a unique system, with available genetic tools, to examine host-pathogen interactions, plant-fungus mutualistic relationships, and fungal development. This commentary highlights overlooked pathogenic and mutualistic aspects of B. bassiana that assist this fungus in shifting along the saprobe/parasite/mutualist continuum. Addressing these knowledge gaps and scrutinizing valuable players driving such a spectrum of ecological interactions will enrich our knowledge of fundamental environmental microbiology and help develop new approaches to pest control and sustainable farming.

## COMMENTARY

**FIG 1:**
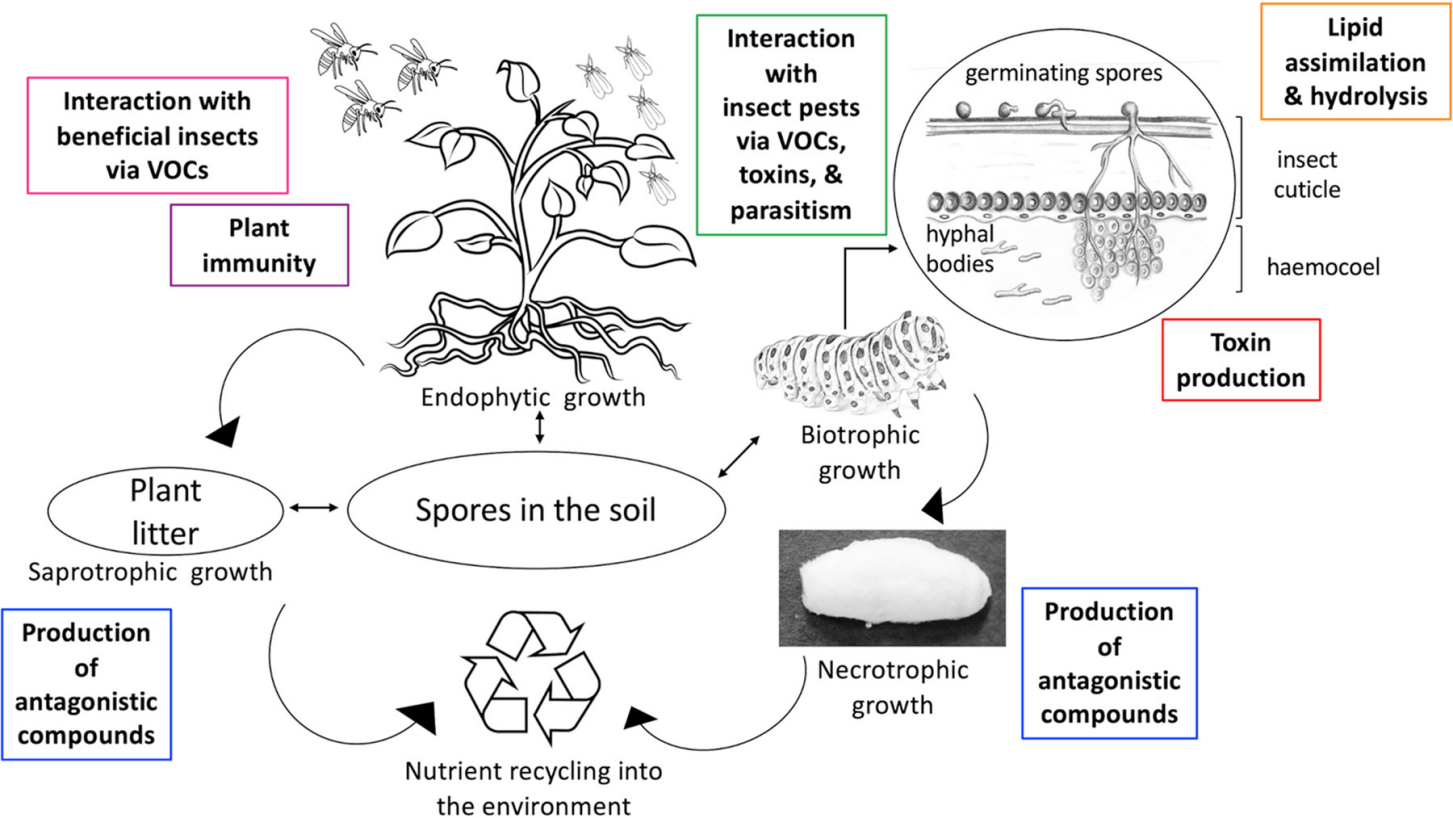
The transition of Beauveria bassiana along the saprobe/mutualist/parasite continuum. B. bassiana is naturally present in the soil, where it lives as a saprotroph exploiting leaf litter and other organic matter for energy and nutrients. As a mutualist, B. bassiana establishes associations with plants by growing as an endophyte (i.e., growing without inducing drastic changes in plant immunity or causing harm to the plant). In exchange for carbon, B. bassiana benefits its host plants in varied ways, including protecting them from insect pests. B. bassiana-mediated resistance toward insects in plants may encompass direct parasitism and production of toxins and volatile organic compounds (VOCs), deterring insect pests, or interacting with beneficial insects. B. bassiana is a generalist insect pathogen. Spores attach to the insect cuticle, germinating and penetrating the insect exoskeleton and invading and proliferating in the hemocoel. Lipid assimilation and hydrolysis are central for this fungus to breach the insect cuticle. As a parasite, B. bassiana switches from biotrophic to a necrotrophic growth (growing and sporulating on the cadaver). Production of toxins during infection may accelerate the death of the insect and signal the biotrophic-necrotrophic switch. Also, some toxic proteins/metabolites could antagonize microbial competitors during necrotrophic/saprophytic growth.

Entomopathogenic fungi are significant factors in natural insect epizootic infections worldwide. Particularly, Beauveria bassiana can infect more than 700 insect species from all the major insect orders (1). Like other insect-pathogenic fungi, B. bassiana penetrates the insect integument and makes its way toward the insect hemocoel, where it proliferates and causes the death of the hosts before growing outward and sporulating on the cadaver (2, 3) (Fig. 1). B. bassiana also grows as an endophyte, establishing mutualistic associations with a wide array of plants (3). The wide-host-range nature and varied lifestyles of B. bassiana, coupled to readily available genomic and molecular tools, provide a tractable system to (i) examine the drivers of fungal evolution, distribution, parasitism, and mutualism and (ii) develop novel approaches for sustainable pest control and farming.

Although significant progress has been made in our understanding of the fundamental and applied aspects of this fungus, this commentary addresses facets of B. bassiana pathogenesis and mutualism that remained understudied and yet are critical for this fungus to operate in the saprobe/parasite/mutualist continuum (Fig. 1). These include lack of basic information (genetic mechanisms) on the nature of the endophytic association and mechanisms driving the success of B. bassiana as a broad-host-range insect pathogen, such as the ability of B. bassiana to assimilate, detoxify, and hydrolyze a wide range of lipids, and the capacity to produce (putative) toxins. While lipid assimilation/detoxification/hydrolysis is known to contribute to the ability of B. bassiana to overcome the antimicrobial barrier of the insect cuticle (i.e., waxy layer), the production of toxins may mediate (i) transitions during host infection and death (i.e., biotrophic to necrotrophic growth), (ii) transitions during rhizosphere interactions and endophyte establishment and persistence, and (iii) antagonism toward microbial competitors. Understanding the mutualistic lifestyle of B. bassiana remains a nascent field in the development of hypotheses on plant-fungus interactions, including resistance to biotic/abiotic stresses and plant growth promotion. Our laboratory currently examines the molecular mechanisms underlying B. bassiana endophytic growth and B. bassiana-mediated plant resistance toward insects to gain a fundamental understanding of these processes and to exploit B. bassiana applications better.

## INSECT PATHOGENESIS AND VIRULENCE: ASSIMILATION AND HYDROLYSIS OF LIPIDS AND SECRETION OF TOXINS

To infect the insect, B. bassiana needs to adhere to the insect epicuticle, a hydrophobic layer consisting of lipids and other compounds, some of which have potent antimicrobial activities (e.g., *n*-alkanes, fatty acids, and aldehydes). Much of the success of B. bassiana as an insect pathogen relies in its ability to overcome this antimicrobial barrier. B. bassiana contains a subset of cytochrome P450 monooxygenases which allows it to use (potentially antimicrobial) lipids as a sole source of carbon, generating fatty acyl coenzyme As (acyl-CoAs) for membranes, lipid storage, or β-oxidation (4). Assimilation of lipids by B. bassiana (and other pathogenic fungi) increases lipid droplet formation and lipid mobilization, which have been linked to virulence (i.e., production of penetration structures and priming of more infectious spores) and to greater stress tolerance and spore dispersal (5, 6).

Molecular analyses of host cuticular lipid hydrolysis by insect-pathogenic fungi remains scarce, despite being recognized as one of the major mechanisms utilized by these fungi to penetrate the cuticle (along with chitin and protein hydrolysis). One limitation to pursuing this line of research is the presence of multiple lipases (e.g., 43 lipase genes identified in B. bassiana [7]) with potential overlapping or redundant activities. Further complicating these efforts is the variation in cuticular lipids across B. bassiana hosts, making it possible that a lipase gene may be central for virulence toward one host but not another. Application of comparative genomics and construction of CRISPR Cas9-based multiple knockout mutants can help unveil the role of lipases in B. bassiana pathogenesis, virulence, development, and fitness, along with the likely need to test multiple different insect hosts.

After breaching the insect cuticle and reaching the hemocoel, the fungus undergoes a dimorphic transition to grow as yeast-like cells, termed hyphal bodies (Fig. 1). These cells can evade the host immune system to colonize insect organs and can secrete toxic proteins that elicit melanization and tissue necrosis (8, 9). Although some of these toxins may be strain specific, potentially contributing to the phenomenon of strain hypervirulence, the identification of genes encoding these toxins is challenging due to difficulties associated with the purification of native protein from crude extracts (e.g., low yields and loss of activity) and often low homology to known toxins. To date, there is no clear picture of the role of these (putative) protein toxins in pathogenesis or plant association, as gene targets have yet to be identified and functional studies are lacking.

Although several B. bassiana bacterial toxin-like (putative) toxins, including seven Cry protein-like toxins, 13 heat-labile enterotoxins, and three zeta toxins, have been identified in genomic analyses (7), little is known about whether these are functional toxins and, if so, how they participate across B. bassiana’s lifestyles. Although the genes are found in other fungal entomopathogens, B. bassiana appears particularly rich in these genes. Our current hypotheses are that Cry-like toxins and enterotoxins may alter insect gut epithelial permeability, facilitating noncanonical routes (as opposed to the cuticle penetration route) of infection via the gut after the ingestion of fungal spores. Alternatively, zeta toxins may not have direct effects in fungal infection and could act as bactericidal agents during fungal necrotrophic/saprophytic growth, similar to what has been reported for the B. bassiana secondary metabolite oosporein (10). Genetic dissection of B. bassiana toxins will shed light on fungal mechanisms of acquisition of hypervirulence, alternative routes of pathogenesis, and fungal adaptations to antagonize microbial competitors.

## ENDOPHYTIC GROWTH: ENDOPHYTIC ESTABLISHMENT AND ENDOPHYTE-MEDIATED INSECT PEST RESISTANCE

Knowledge on the molecular basis mediating B. bassiana plant colonization remains minimal. Within the context of host-pathogen interaction, the greater the number of spores that attach, germinate, and penetrate the cuticle, the faster the fungus colonizes the insect hemocoel and causes death. However, extrapolation to microbe-plant association may be misleading. When microbes attempt to colonize plants, they are recognized by the plant immune system, including the salicylic acid (SA) pathway. Plant SA alters the composition and assembly of the plant microbiome, and plants expressing constitutive production of SA show slower fungal colonization (11, 12). Low spore attachment can result in reduced penetration of the root tissue, causing minimal activation of the SA pathway and potentially allowing for more robust endophyte establishment by B. bassiana. A comprehensive examination of plant immune activation upon B. bassiana colonization (i.e., the SA-dependent systemic acquired resistance) is needed and will provide critical information on B. bassiana growth dynamics within the host plants. Furthermore, like other fungal endophytes, B. bassiana has the potential to bypass the activation of plant SA-dependent systemic acquired resistance by producing a (fungal) salicylate hydrolase. This enzyme potentially would enable the fungus to grow endophytically without causing drastic changes in plant immunity.

**FIG 2:**
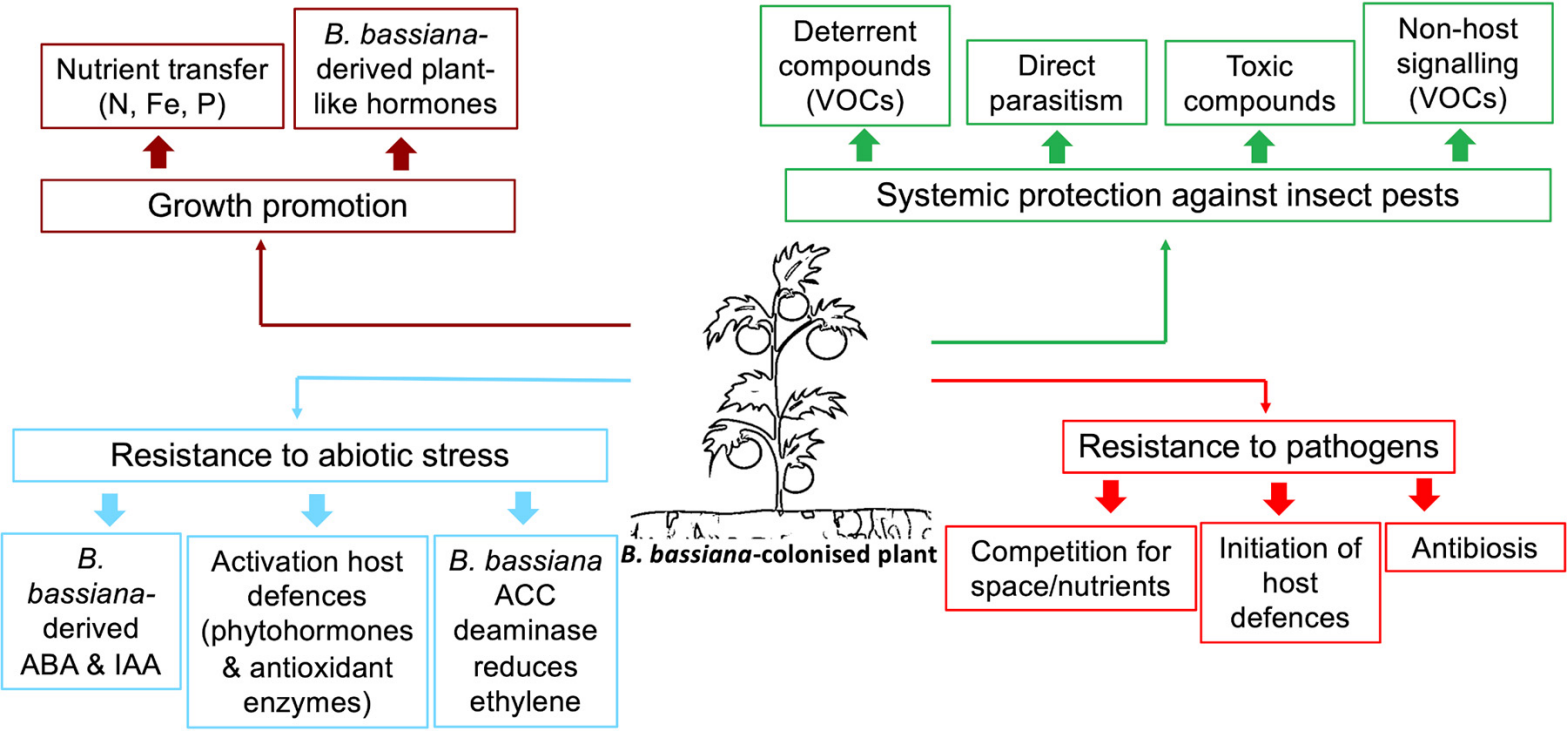
Proposed mechanisms mediating the benefits of plant-Beauveria bassiana association. Growth promotion (brown boxes): enhanced nutrient transfer, for example, nitrogen (N), phosphorus (P), and iron (Fe) (13, 14), could reduce fertilizer usage, offering savings and reducing greenhouse emissions. Resistance to insect pests and pathogens (green and red boxes) could reduce the need for fungicide and insecticide use, thereby alleviating their environmental and health burdens. B. bassiana may increase plant tolerance to abiotic stresses (blue boxes) via the activation of host stress responses and the production of antioxidant enzymes (15). Osmotic stress upregulates the levels of abscisic acid (ABA), auxin (indole-3-acetic-acid [IAA]), and ethylene, mediating stomatal closure, expression of stress resistance genes, and production of antioxidant enzymes (16, 17). ACC (aminocyclopropane-1-carboxylic) deaminase regulates excessive ethylene levels in plants by cleaving the ethylene precursor ACC into ammonia and α-ketobutyrate, preventing arrested plant growth, chlorosis, senescence, and death (18). VOCs, volatile organic compounds.

Unique and understudied aspects of endophytic colonization by B. bassiana are (i) any impacts on arthropod pests feeding on colonized plants, (ii) the extent to which B. bassiana may safeguard plants from plant pathogens, and (iii) the degree and conditions driving growth promotion and increased plant abiotic stress tolerances (Fig. 2). While several studies have shown these various benefits, little is known concerning the underlying genetic and biochemical mechanisms involved. Although some attention has been directed toward examining the potential protective effects of endophytic B. bassiana against insect pests, this research remains limited and often shows contradictory results. Current hypotheses include that B. bassiana might protect plants from phytophagous arthropods directly (e.g., parasitism or production of toxic compounds) and/or indirectly by deterring arthropods from ovipositing on or colonizing the plant. B. bassiana can be found as a natural epiphyte on many plants, which supports potential direct parasitism via topical acquisition of fungal spores from the plant canopy. Alternatively, Cry-like toxins and enterotoxins could be produced *in planta* during fungal endophytic growth, providing plants with direct systemic protection against insect pests, an idea we are currently exploring.

One of the major gaps in our understanding of the fungus-plant-insect interaction is the role(s) of communication signals arbitrated by volatile organic compounds (VOCs). Fungal endophytes can release VOCs causing major changes in the plant chemistry. However, it is not clear if B. bassiana and/or B. bassiana-induced plant VOCs attract or deter arthropod colonization. Potentially, these VOCs may enable herbivore and nonhost signaling (i.e., recruiting enemies of herbivorous insects). By attracting insects, VOCs could benefit the fungus by providing hosts and potentially even benefit the plant via nitrogen transfer from fungus-mediated insect mortality (13), or by recruiting parasitoids that attack herbivores. Conversely, VOCs could result in insect deterrence and priming of antiherbivore responses in noncolonized plants via plant-to-plant communication via airborne or soilborne VOCs. As little is known in this regard, elucidating the underlying chemistry and molecular basis of VOCs within these interactions is an exciting novel research direction.

## THE PATHWAY FORWARD: IDENTIFYING ESSENTIAL PLAYERS INVOLVED IN PATHOGENESIS AND PLANT-FUNGUS INTERACTION

Identifying the main participants in B. bassiana parasitic and mutualistic interactions requires a more complete insight into the cross talk between B. bassiana, insect host, and plant partners. In this regard, combined “omics” analyses (e.g., transcriptomics, epigenomics, and proteomics) of fungus-challenged insects and plants can provide simultaneous profiles of the nature of these interactions. Plants with low genetic redundancy, e.g., liverworts, could represent simpler models than other land plant systems to dissect the molecular basis of the fungus-plant interaction. Cell biological (determining where in the plant) and genetic (gene knockout) approaches can provide more nuanced understanding of the mechanistic bases underpinning fungal infection and mutualism. Finally, knowledge gain from these studies can be translated into important applications impacting food safety and security, biodiversity, and crop yield and resilience.

## References

[1] Ortiz-Urquiza A, Keyhani NO. 2016. Molecular genetics of *Beauveria bassiana* infection of insects. Adv Genet 94:165–249. doi:10.1016/bs.adgen.2015.11.003. 27131326

[2] Ortiz-Urquiza A, Keyhani ON. 2013. Action on the surface: entomopathogenic fungi versus the insect cuticle. Insects 4:357–374. doi:10.3390/insects4030357. 26462424 PMC4553469

[3] Ortiz-Urquiza A, Luo Z, Keyhani NO. 2015. Improving mycoinsecticides for insect biological control. Appl Microbiol Biotechnol 99:1057–1068. doi:10.1007/s00253-014-6270-x. 25503318

[4] Pedrini N, Ortiz-Urquiza A, Huarte-Bonnet C, Zhang S, Keyhani NO. 2013. Targeting of insect epicuticular lipids by the entomopathogenic fungus *Beauveria bassiana*: hydrocarbon oxidation within the context of a host-pathogen interaction. Front Microbiol 4:24. doi:10.3389/fmicb.2013.00024. 23422735 PMC3573267

[5] Fan Y, Ortiz‐Urquiza A, Garrett T, Pei Y, Keyhani NO. 2015. Involvement of a caleosin in lipid storage, spore dispersal, and virulence in the entomopathogenic filamentous fungus, *Beauveria bassiana*. Environ Microbiol 17:4600–4614. doi:10.1111/1462-2920.12990. 26235819

[6] Ortiz-Urquiza A, Fan Y, Garrett T, Keyhani NO. 2016. Growth substrates and caleosin-mediated functions affect conidial virulence in the insect pathogenic fungus *Beauveria bassiana*. Microbiology (Reading) 162:1913–1921. doi:10.1099/mic.0.000375. 27655425

[7] Xiao G, Ying S-H, Zheng P, Wang Z-L, Zhang S, Xie X-Q, Shang Y, St Leger RJ, Zhao G-P, Wang C, Feng M-G. 2012. Genomic perspectives on the evolution of fungal entomopathogenicity in *Beauveria bassiana*. Sci Rep 2:483. doi:10.1038/srep00483. 22761991 PMC3387728

[8] Ortiz-Urquiza A, Riveiro-Miranda L, Santiago-Álvarez C, Quesada-Moraga E. 2010. Insect-toxic secreted proteins and virulence of the entomopathogenic fungus *Beauveria bassiana*. J Invertebr Pathol 105:270–278. doi:10.1016/j.jip.2010.07.003. 20674578

[9] Ortiz-Urquiza A, Keyhani NO, Quesada-Moraga E. 2013. Culture conditions affect virulence and production of insect toxic proteins in the entomopathogenic fungus *Metarhizium anisopliae*. Biocontrol Sci Technol 23:1199–1212. doi:10.1080/09583157.2013.822474.

[10] Fan Y, Liu X, Keyhani NO, Tang G, Pei Y, Zhang W, Tong S. 2017. Regulatory cascade and biological activity of Beauveria bassiana oosporein that limits bacterial growth after host death. Proc Natl Acad Sci USA 114:E1578–E1586. doi:10.1073/pnas.1616543114. 28193896 PMC5338512

[11] Lebeis SL, Paredes SH, Lundberg DS, Breakfield N, Gehring J, McDonald M, Malfatti S, Glavina del Rio T, Jones CD, Tringe SG, Dangl JL. 2015. Salicylic acid modulates colonization of the root microbiome by specific bacterial taxa. Science 349:860–864. doi:10.1126/science.aaa8764. 26184915

[12] Martínez-Medina A, Appels FVW, van Wees SCM. 2017. Impact of salicylic acid- and jasmonic acid-regulated defences on root colonization by *Trichoderma harzianum* T-78. Plant Signal Behav 12:e1345404. doi:10.1080/15592324.2017.1345404. 28692334 PMC5616143

[13] Behie SW, Zelisko PM, Bidochka MJ. 2012. Endophytic insect-parasitic fungi translocate nitrogen directly from insects to plants. Science 336:1576–1577. doi:10.1126/science.1222289. 22723421

[14] Sánchez-Rodríguez AR, Raya-Díaz S, Zamarreño ÁM, García-Mina JM, del Campillo MC, Quesada-Moraga E. 2018. An endophytic *Beauveria bassiana* strain increases spike production in bread and durum wheat plants and effectively controls cotton leafworm (Spodoptera littoralis) larvae. Biol Control 116:90–102. doi:10.1016/j.biocontrol.2017.01.012.

[15] Lata R, Chowdhury S, Gond SK, White JF. 2018. Induction of abiotic stress tolerance in plants by endophytic microbes. Lett Appl Microbiol 66:268–276. doi:10.1111/lam.12855. 29359344

[16] Dolferus R. 2014. To grow or not to grow: a stressful decision for plants. Plant Sci 229:247–261. doi:10.1016/j.plantsci.2014.10.002. 25443851

[17] Bielach A, Hrtyan M, Tognetti VB. 2017. Plants under stress: involvement of auxin and cytokinin. Int J Mol Sci 18:1427. doi:10.3390/ijms18071427. PMC5535918 28677656

[18] Gamalero E, Glick BR. 2015. Bacterial modulation of plant ethylene levels. Plant Physiol 169:13–22. doi:10.1104/pp.15.00284. 25897004 PMC4577377

